# Complete genome sequence of *Sporomusa sphaeroides* DSM 2875^T^ isolated from mud of the Leine river and *Sporomusa ovata* DSM 2662^T^ isolated from sugar beet leaf silage

**DOI:** 10.1128/mra.00372-24

**Published:** 2024-07-30

**Authors:** Tim Böer, Alina Lüschen, Rolf Daniel, Anja Poehlein

**Affiliations:** 1 Genomic and Applied Microbiology and Göttingen Genomics Laboratory, Institute of Microbiology and Genetics, Georg-August University of Göttingen, Göttingen, Germany; Rochester Institute of Technology, Rochester, New York, USA

**Keywords:** *Sporomusa sphaeroides*, *Sporomusa ovata*, Wood-Ljungdahl pathway, acetogenic bacteria, *Sporomusa*

## Abstract

We report the closed genome sequences of the acetogen *Sporomusa sphaeroides* E^T^ (DSM 2875^T^) and of *Sporomusa ovata* H1^T^ (DSM 2662^T^). The *S. sphaeroides* E^T^ genome harbors a chromosome (4,956,256 bp) and a plasmid (59,087 bp). The genome of *S. ovata* H1^T^ harbors one chromosome (5,433,971 bp).

## ANNOUNCEMENT

*Sporomusa* is an exclusively acetogenic genus, which grows autotrophically with H_2_ + CO_2_ and produces acetate as sole fermentation product. *Sporomusa* members attracted attention as efficient electrotrophic biocatalysts employed in microbial electrosynthesis for the production of biocommodities such as acetate or biofuels, simultaneously fixing the greenhouse gas CO_2_ (1, 2). However, the only currently available complete *Sporomusa* genome sequence is from *Sporomusa termitida* (3). We report the complete genome sequences of the *Sporomusa* type species *Sporomusa sphaeroides* E^T^ (DSM 2875^T^) isolated from mud of the Leine river (Göttingen, Germany) and the type strain of *Sporomusa ovata* H1^T^ (DSM 2662^T^) isolated from sugar beet leaf silage (Göttingen, Germany) (4).

Both strains were cultivated in 10 mL of DSM 311c medium as listed by the German Collection of Microorganisms and Cell Cultures (DSMZ, Braunschweig, Germany) under anaerobic conditions. Cells were inoculated from lyophilized stock cultures from the DSMZ in an anaerobic chamber and incubated in Hungate tubes at 35°C for 12 h without shaking. Cells were harvested by centrifugation at 18,000 × *g* for 5 min. For Illumina sequencing and Nanopore sequencing, separately grown cell cultures were used. DNA was isolated by using the Master-Pure Complete DNA and RNA purification kit (Epicentre, Madison, WI, USA) following the instructions for cell samples. Illumina sequencing was performed by the preparation of Illumina sequencing libraries using the Nextera XT DNA sample preparation kit and using a MiSeq system with v3 chemistry (2 × 300 bp, 600 cycles) following the instructions of the manufacturer (Illumina, San Diego, CA, USA). Nanopore sequencing libraries were prepared with 1.5 µg high-molecular-weight DNA using the ligation sequencing kit 1D^2^ (SQK-LSK308) for *S. sphaeroides* E^T^ and the ligation sequencing kit 1D (SQK-LSK108) for *S. ovata* H1^T^ as well as the native barcode expansion kit (EXP-NBD103) as recommended by the manufacturer (Oxford Nanopore Technologies, Oxford, UK). Nanopore sequencing was conducted for 72 h using the MinION device Mk1B, a SpotON flow cell R9.4.1 and the MinKNOW software (v1.10.11) as recommended by the manufacturer (Oxford Nanopore Technologies). Default parameters were used for all software unless otherwise specified. Base calling of Nanopore sequencing data was performed with the Albacore software (v2.0.1).

**Fig 1:**
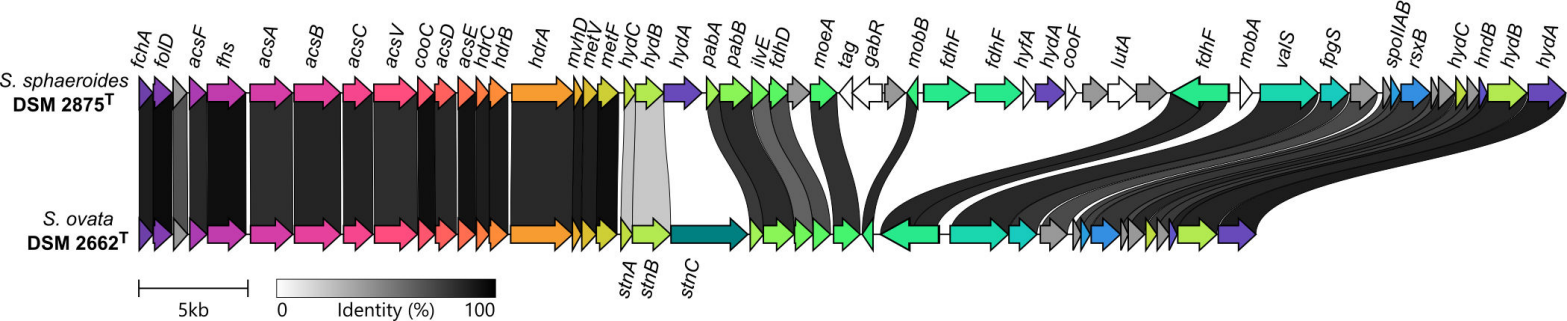
Comparison of the Wood-Ljungdahl cluster of *S. sphaeroides* DSM 2875^T^ and *S. ovata* DSM 2662^T^. The following gene abbreviations were used: *fchA*, methenyl THF cyclohydrolase; *folD*, bifunctional cyclohydrolase/dehydrogenase; *acsF*, carbon monoxide dehydrogenase accessory protein; *fhs*, formyl THF synthetase; *acsA*, anaerobic carbon-monoxide dehydrogenase catalytic subunit; *acsB*, carbon monoxide dehydrogenase/acetyl-CoA synthase subunit beta; *acsC*, CoFeSP large subunit; *acsV*, corrinoid activation/regeneration protein; *cooC*, carbon monoxide dehydrogenase accessory protein; *acsD*, CoFeSP small subunit; *acsE*, methyl THF CoFeSP methyltransferase; *hdrC*, heterodisulfide oxidoreductase iron-sulfur cluster-binding subunit; *hdrB*, heterodisulfide reductase subunit B; *hdrA*, heterodisulfide reductase subunit A; *mvhD*, methyl-viologen-reducing hydrogenase delta subunit; *metV*, methylene THF reductase C-terminal catalytic subunit; *metF*, methylene THF reductase large subunit; *hydC*, electron bifurcating hydrogenase subunit HydC; *hydB*, electron bifurcating hydrogenase subunit HydB; *hydA*, electron bifurcating hydrogenase subunit HydA; *pabA*, aminodeoxychorismate/anthranilate synthase component 2; *pabB*, aminodeoxychorismate synthase component 1; *ilvE*, branched-chain-amino-acid aminotransferase; *fdhD*, sulfur carrier protein; *moeA*, molybdopterin molybdenumtransferase; *tag*, DNA-3-methyladenine glycosylase 1; *gabR*, HTH-type transcriptional regulatory protein GabR; *mobB*, molybdopterin-guanine dinucleotide biosynthesis adapter protein; *fdhF*, formate dehydrogenase H; *hyfA*, hydrogenase-4 component A; *cooF*, iron-sulfur protein; *lutA*, lactate utilization protein A; *mobA*, molybdenum cofactor guanylyltransferase; *valS*, valine--tRNA ligase; *fpgS*, folylpolyglutamate synthase; *spoIIAB*, anti-sigma F factor; *rsxB*, ion-translocating oxidoreductase complex subunit B; *hndB*, NADP-reducing hydrogenase subunit HndB; *stnA*, *Sporomusa*-type Nfn transhydrogenase subunit A; *stnB*, *Sporomusa*-type Nfn transhydrogenase subunit B; *stnC*, *Sporomusa*-type Nfn transhydrogenase subunit C.

The following programs were used for genome assemblies: Trimmomatic (v0.36; LEADING: 3, TRAILING: 3, SLIDINGWINDOW:4:15, MINLEN:50) (5) and Unicycler (v0.4.0) (6). The Unicycler pipeline was used for the combined assembly of the Nanopore and the Illumina reads resulting in circular chromosomes for both genomes. Identification and trimming of the plasmid overlap was performed as part of the standard Unicycler pipeline. Genome annotations were performed with Prokka (v1.14.5) (7) and PGAP (v6.6) (8), and quality assessment of the final genome assemblies was conducted with CheckM2 (v1.0.2) (9). Protein-encoding genes containing selenocysteine (Sec) and pyrrolysine (Pyl) residues were manually identified and annotated. Details of sequencing and genome statistics of *S. sphaeroides* E^T^ and *S. ovata* H1^T^ are summarized in Table 1. The comparison of the Wood-Ljungdahl gene cluster of both strains (Fig. 1) was visualized with Clinker (v0.0.28) (10).

**TABLE 1:** Sequencing statistics and genome features of *S. sphaeroides* E^T^ and *S. ovata* H1^T^

Feature	*S. sphaeroides* E^T^ (DSM 2875^T^)	*S. ovata* H1^T^ (DSM 2662^T^)
Chromosome size (bp)	4,956,256	5,433,971
Number of Illumina reads (250 bp)	4,388,306	2,924,484
Number of Nanopore reads/mean length (bp)	50,628/3,746	50,548/3,260
Nanopore reads N_50_ (bp)	6,797	5,646
Chromosome mean coverage (Illumina/Nanopore)	232/27	145/30
Plasmid size (bp)	59,087	–^*a*^
Plasmid mean coverage (Illumina/Nanopore)	269/35	–
GC content (%)	47	43
Genes	4,656	5,314
CDS	4,511	5,112
Functional proteins	2,596	3,609
Hypothetical proteins	1,915	1,503
rRNAs (5S, 16S, 23S)	33 (10, 12, 11)	42 (15, 15, 12)
tRNAs	111	160
tmRNAs	1	1
CheckM2:		
Completeness score (%)	99.99	100
Contamination score (%)	1.19	7.57

^*a*^ "–", not present.

## Data Availability

Genome sequences were deposited under the GenBank accession numbers CP146991 and CP146992 (*S. sphaeroides*) and CP146301 (*S. ovata*). Raw read data were deposited in the NCBI Sequence Read Archive (SRA) under the accession numbers SRR28314578 (Illumina) and SRR28314177 (Nanopore) for *S. sphaeroides,* and SRR28327341 (Illumina) and SRR28327318 (Nanopore) for *S. ovata*.

## References

[1] Madjarov J, Soares R, Paquete CM, Louro RO. 2022. Sporomusa ovata as catalyst for bioelectrochemical carbon dioxide reduction: a review across disciplines from microbiology to process engineering. Front Microbiol 13:913311. doi:10.3389/fmicb.2022.913311 35801113 PMC9253864

[2] Thulluru LP, Ghangrekar MM, Chowdhury S. 2023. Progress and perspectives on microbial electrosynthesis for valorisation of CO_2_ into value-added products. J Environ Manage 332:117323. doi:10.1016/j.jenvman.2023.117323 36716542

[3] Poehlein A, Hollensteiner J, Dreyer A, Gavrilova I, Daniel R. 2020. Complete genome sequence of Sporomusa termitida DSM 4440^T^. Microbiol Resour Announc 9:e00046-20. doi:10.1128/MRA.00046-20 32165380 PMC7067948

[4] Möller B, Oßmer R, Howard BH, Gottschalk G, Hippe H. 1984. Sporomusa, a new genus of gram-negative anaerobic bacteria including Sporomusa sphaeroides spec. nov. and Sporomusa ovata spec. nov. Arch. Microbiol 139:388–396. doi:10.1007/BF00408385

[5] Bolger AM, Lohse M, Usadel B. 2014. Trimmomatic: a flexible trimmer for Illumina sequence data. Bioinformatics 30:2114–2120. doi:10.1093/bioinformatics/btu170 24695404 PMC4103590

[6] Wick RR, Judd LM, Gorrie CL, Holt KE. 2017. Unicycler: resolving bacterial genome assemblies from short and long sequencing reads. PLoS Comput Biol 13:e1005595. doi:10.1371/journal.pcbi.1005595 28594827 PMC5481147

[7] Seemann T. 2014. Prokka: rapid prokaryotic genome annotation. Bioinformatics 30:2068–2069. doi:10.1093/bioinformatics/btu153 24642063

[8] Tatusova T, DiCuccio M, Badretdin A, Chetvernin V, Nawrocki EP, Zaslavsky L, Lomsadze A, Pruitt KD, Borodovsky M, Ostell J. 2016. NCBI prokaryotic genome annotation pipeline. Nucleic Acids Res 44:6614–6624. doi:10.1093/nar/gkw569 27342282 PMC5001611

[9] Chklovski A, Parks DH, Woodcroft BJ, Tyson GW. 2023. CheckM2: a rapid, scalable and accurate tool for assessing microbial genome quality using machine learning. Nat Methods 20:1203–1212. doi:10.1038/s41592-023-01940-w 37500759

[10] Gilchrist CLM, Chooi Y-H. 2021. clinker &amp; clustermap.js: automatic generation of gene cluster comparison figures. Bioinformatics 37:2473–2475. doi:10.1093/bioinformatics/btab007 33459763

